# Shear strength and particle breakage of construction and demolition waste as a function of moisture state and compaction level: Insights for sustainable highway engineering

**DOI:** 10.1371/journal.pone.0298765

**Published:** 2024-03-29

**Authors:** Ahmed M. Yosri, Abdelhalim Azam, Fayez Alanazi, Abdulaziz H. Alshehri, Mohamed Ahmed Okail

**Affiliations:** 1 Civil Engineering Department, Jouf University, Sakaka, KSA; 2 Civil Engineering Department, Delta University for Science and Technology, Dakahlia Governorate, Egypt; 3 Public Works Engineering Department, Faculty of Engineering, Mansoura University, Dakahlia Governorate, Egypt; 4 Department of Civil Engineering, College of Engineering, Najran University, Najran, Saudi Arabia; 5 Faculty of Engineering and Technology, Badr University in Cairo, Cairo, Egypt; University of Duhok, IRAQ

## Abstract

In this study, the variation of shear strength behavior and particle breakage (after shearing), as a function of moisture state and compaction level, is investigated for recycled concrete aggregate blended with recycled clay masonry. Recycled masonry was blended with concrete aggregate in percentages ranging from 0% to 30% by total weight. Tests include; basic engineering characteristics (particle size, modified compaction, hydraulic conductivity, and California Bearing Ratio, CBR) as well as unconsolidated undrained static triaxial testing. In triaxial tests, moisture levels ranged from 60% to 100% of optimum moisture content, but compaction levels ranged from 90% to 98% of maximum dry density. The hydraulic conductivity for blends is approximately 2x10^-6^ cm/s, which indicates a relatively low hydraulic conductivity. Results show a proportional linear relationship between the shear strength of blends and the level of compaction. Despite this, both apparent cohesion and shear strength exhibited reverse linear trends. As expected, more compaction effort resulted in more particle breakage. Strict control should be performed over the compaction process to achieve the required compaction level which resulting in pavement materials being stiffer.

## 1. Introduction

The construction industry consumes a large amount of natural aggregate and it is well-known that the natural resources are limited and demand considerable energy consumption in the production stage, resulting in substantial emissions and environmental impacts [1–3]. Nowadays, most developed countries enforce most research interests to find out suitable alternative materials. Due to the rehabilitation, maintenance, and reconstruction process of many structures, large amount of waste materials are produced. One of the possible ways to decrease the environmental impact of the exploration of natural aggregate is finding a feasible application of those waste materials in pavement construction. The employment of waste materials in the construction industry solves the disposal problem of those materials [4–7].

The construction and demolition (C&D) waste consists mainly of concrete aggregate, masonry, wood, and some other materials. Therefore, there is much interest in recycling and producing alternative uses of waste. Several waste materials can be used as a pavement material such as recycled concrete aggregate, waste rocks, waste foundry sand, crushed glass, reclaimed asphalt pavement (RAP), and crushed masonry have been shown [1, 8–14].

The application of C&D waste in construction is beneficial and sustainable, as it reduces both the request for natural quarry material and undesirable waste. Furthermore, less landfill area is needed and decreases the energy costs for the production process of quarry materials [15]. Hossain et al., [16] evaluated the feasibility of using C&D as alternatives to natural aggregates by life cycle assessment. It was concluded that the use of recycled aggregate in pavement construction reduces greenhouse gases emission and energy consumption by 65% and 58%, respectively compared with natural aggregate. It is also reported that pavement life increases and global warming is reduced by 20% in case of using recycled materials [17].

Saberian et al., [18] and, Saberian et al., [19] investigated the shear behaviour of recycled waste materials (recycled concrete aggregate) blended with the recycled tire as pavement construction materials (base/subbase). The effect of size and the amount of recycled tire on the shear strength parameters were evaluated. It was found that the addition of fine and coarse aggregates of crumb rubber has increased the cohesion of the blends. In a similar study, Perera et al., [20] assessed the geotechnical properties of two types of C&D materials, blended with 5% shredded Polyethylene terephthalate (PET) plastic. The authors found that the addition of 5%PET improved the shear strength parameters for the C&D waste. Arulrajah et al. [2] assessed the shear strength properties of a range of recycled materials. The materials were recycled concrete aggregate, crushed brick, reclaimed asphalt pavement, waste excavation rock, fine recycled glass and medium recycled glass. The authors found that the recycled materials meet the shear strength requirements for the application as subbase and base in pavement construction.

The engineering characteristics of unbound granular materials (UGMs) (base and subbase) are highly affected by the degree of compaction and the existing moisture state [1, 14, 21, 22]. Furthermore, the pavement layers are located above the groundwater table and remain in the unsaturated zone during the pavement life. Consequently, the variations in moisture that occurs due to the change in environmental states affect the engineering properties of the pavement materials [23, 24].

Shear strength parameters, such as the cohesion and friction angle, characterize a material’s ability to resist deformation under applied loads. In the context of pavements, rutting occurs when the pavement material deforms and forms depressions due to traffic-induced stresses. Higher shear strength values indicate a greater resistance to deformation, and better support the traffic loads without undergoing excessive deformation, which is essential for preventing rutting [25, 26]. Reducing rutting is essential for minimizing maintenance and rehabilitation costs. Pavements that are prone to rutting require more frequent repairs and maintenance, which can be costly. Therefore, optimizing shear strength parameters in pavement materials can contribute to cost savings over the life of the pavement [27].

The shear strength of recycled materials in pavement design is of paramount importance as it directly affects the structural integrity, durability, and overall performance of pavements constructed with such materials. However, understanding and optimizing the shear strength of these materials is crucial to maximize the environmental benefits and enables cost-effective pavement design and construction while maintaining structural performance [28].

In modern pavement design methods, such as the Mechanistic-Empirical Pavement Design Guide (MEPDG) (ASHHTO 2015) [29], shear strength parameters are integrated into the design process to improve the accuracy of pavement performance predictions.

The absence of studies investigating the variations of shear strength with moisture content and compaction level, particularly for recycled materials, underscores an important research gap in the field of geotechnical and pavement engineering. Here are some reasons why such research is needed.

- Understanding how these factors influence shear strength is essential for the effective use of recycled materials in construction.
- Shear strength is a critical parameter for predicting the performance and stability of pavement layers. Investigating its variations with moisture content and compaction level in recycled materials will enable more accurate performance predictions.
- Understanding how moisture and compaction affect shear strength allows for the optimization of material usage. This can lead to more cost-effective and resource-efficient pavement design.
- Research in this area can help establish quality control procedures for recycled materials. Therefore this study is aimed to:
  1. Investigate the moisture sensitivity of shear strength parameters of recycled materials prepared at different moisture levels.
  2. Evaluate the influence of compaction level on shear strength parameters and investigate the effect of static loading on particle breakage.

## 2. Materials and methods

Two products from different suppliers (A and B) were investigated in this study. Supplier A has two different types of recycled material (recycled concrete aggregate and recycled clay brick). The aggregate used for concrete is generally sourced from quarries and consists of materials such as quartzite, dolomite, or siltstone. The brick was blended in the laboratory at three different percentages (10%, 20%, and 30%) with the recycled concrete aggregate. The symbols for the blends were A10, A20, and A30. Supplier B has one pre-mixed blend which consists of 20% recycled brick and 80% recycled concrete aggregate with two different stockpiles. One sample from each stockpile was sourced and named as follows; B20-1 and B20-2.

The laboratory testing program includes grading distribution, consistency limits, water absorption, specific gravity, los angeles abrasion (LAA) test, modified proctor compaction, california bearing ratio (CBR), and the hydraulic conductivity test. The results of those tests were compared with Saudi specifications for road construction, Ministry of Transport, Kingdom of Saudi Arabia, [30].

The performance of the blended materials was evaluated by conducting the unconsolidated undrained triaxial (UU) and therefore determining the undrained shear strength parameters for the different blends. The UU test was conducted at different compaction levels to evaluate the influence of compaction effort on the shear strength parameters. It is known that the moisture content has a significant impact on the durability and permeability of materials. Testing materials across a wide range of moisture levels provides valuable insights into their behavior under various environmental conditions. The Evaluation of material properties at different moisture contents, can in help identifying the optimal moisture content range for the material’s intended use and predicting how the material will perform under varying moisture conditions. It is also helpful in developing strategies to mitigate moisture-related damage and ensure the long-term durability of the material. Therefore, the test was carried out at different initial moisture states (varied from 60% to 100% of optimum moisture content, OMC). Finally, the grading distribution was determined after compaction and shearing to evaluate the particle breakage during the compaction and static loading process. The testing standards used in this study are provided in Table 1.

**Table 1:** Basic engineering characteristics for different blends.

Property / Material		Test Standard	A10	A20	A30	B20-1	B20-2	Saudi Spec.
Passing #200 Sieve, %		AASHTO T 27–99 [37]	7	7	7	6	7	3–10%
Liquid Limit, (%)		AASHTO T 89–96 [38]	23.5	23.0	23.0	27	26	Max. 25%
Plasticity Index, (%)		AASHTO T 90–00 [39]	2.5	2.5	2.0	2.0	0.0	Max. 6%
Los Angeles Abrasion		AASHTO T 96–99 [40]	41	43	45	42	41	Max. 45%
Water absorption		AASHTO T 85–91 [41]	6.2	6.8	7.5	7.4	6.3	Max. 10%
Specific Gravity			2.57	2.55	2.55	2.57	2.56	----
OMC (%)		AASHTO T 180–97 [31]	12.1	12.5	14.2	14.8	11.6	----
MDD (gm/cm^3^)			1.89	1.86	1.84	1.84	1.92	----
Hydraulic conductivity, cm/sec x 10^−6^		ASTM D 5856–95 [32]	2.2	1.8	1.8	20	17	----
CBR (%)	Before Soaking	AASHTO T 193–99 [34]	125	116	83	141	126	Min 65%
	After Soaking		112	101	74	130	115	

To estimate the compaction characteristics (maximum dry density MDD, OMC) for the different blends, a modified Proctor test was carried out according to AASHTO T 180–97 [31].

A series of falling head permeameter tests were conducted in accordance with ASTM D 5856–95 [32] to determine the hydraulic conductivity of the blends. Cylindrical specimens were prepared and compacted at OMC and 98% of MDD dynamically using modified compaction effort in a cylindrical mould having a diameter of 104 mm and height of 115 mm. It worth noting that the compaction process should be conducted directly after wet mixing of the blend as the agglomeration of clay particles at higher moisture content may result in reducing the density and increasing the permeability particularly in soils with a significant clay fraction [33]. The average of all permeability determination was taken as the permeability of the blends.

The CBR is used to evaluate the potential strength of materials and it was performed according to AASHTO T 193–99 [34]. Duplicate specimens were prepared and compacted at the compaction characteristics (OMC and MDD) for each blend using the modified Proctor effort for the different blends. The test was conducted directly on the first specimen after compaction however, the second specimen remained in water (soaked) 4 days and after that, the testing was performed.

To estimate the values of shear strength parameters (apparent cohesion, c, and friction angle, ϕ) for the different blends, a series of unconsolidated-undrained triaxial shear tests were conducted according to AASHTO T 296 [35]. The moisture content of the soil samples was adjusted to the desired moisture states (60%, 70%, 80%, 90%, and 100% OMC) by adding or removing water and it was mixed for a sufficient time to achieve a homogeneous distribution of moisture throughout the soil particles. Cylindrical samples having a diameter of 100 mm and height of 200 mm were prepared at different moisture states ranging from 70% to 90% OMC and at a compaction level of 98% MDD. The A20, B20-1, and B20-2 blends were investigated at two more additional moisture states (60% and 100% OMC) to verify the relationship between peak deviator stress and moisture state.

Additional specimens were prepared at 80% OMC and at three different compaction levels (90%, 95%, and 98% MDD) for material B20-1 to study the influence of compaction level on the shear strength of the recycled materials.

To ensure uniformity of compaction, the static compaction method was selected. A steel mold with a collar and two fitting plungers was used. The mold surfaces were clean and lubricate to prevent sticking and ensure smooth specimen removal. The soil sample was divided into two equal halves then the first half was placed into the mold and compact it using the universal compression machine. Sufficient pressure was applied to achieve the required height for the compacted half and it was maintain for a short duration and then release it. To ensure a good shear interface between the first layer and the final one, a steel rod was used to scratch the surface of the compacted first layer. The compacted specimen was sealed using a plastic bag to prevent moisture loss and the specimen was allowed to cure overnight to ensure uniform moisture distribution and consolidation. Three different confining pressures (25, 50, and 75 kPa) were applied to the tested specimens using appropriate equipment. Confining pressure simulates the stress conditions experienced by soil in various engineering applications. The confining pressures were selected for the recycled materials in this range which are representative of the stress conditions that the recycled materials are likely to encounter in various applications, such as embankments, foundations, and road subgrades [36]. In addition to, recycled materials typically have lower strength than virgin soil materials, so using lower confining pressures is appropriate. A membrane stretcher was used to set the rubber membrane around the specimen then O-rings were used to seal to the end caps of the membrane. A rate of loading of 1.91 mm/min was selected then at the constant confining pressure, a vertical major stress was applied to the specimen and gradually increased till the failure.

Finally, the grading distribution was determined on all blends except the B20-2 blend after the compaction and shearing processes to evaluate the influence of static loading on particle breakage.

## 3. Results and discussion

### 3.1. Basic engineering characteristics

**Fig 1:**
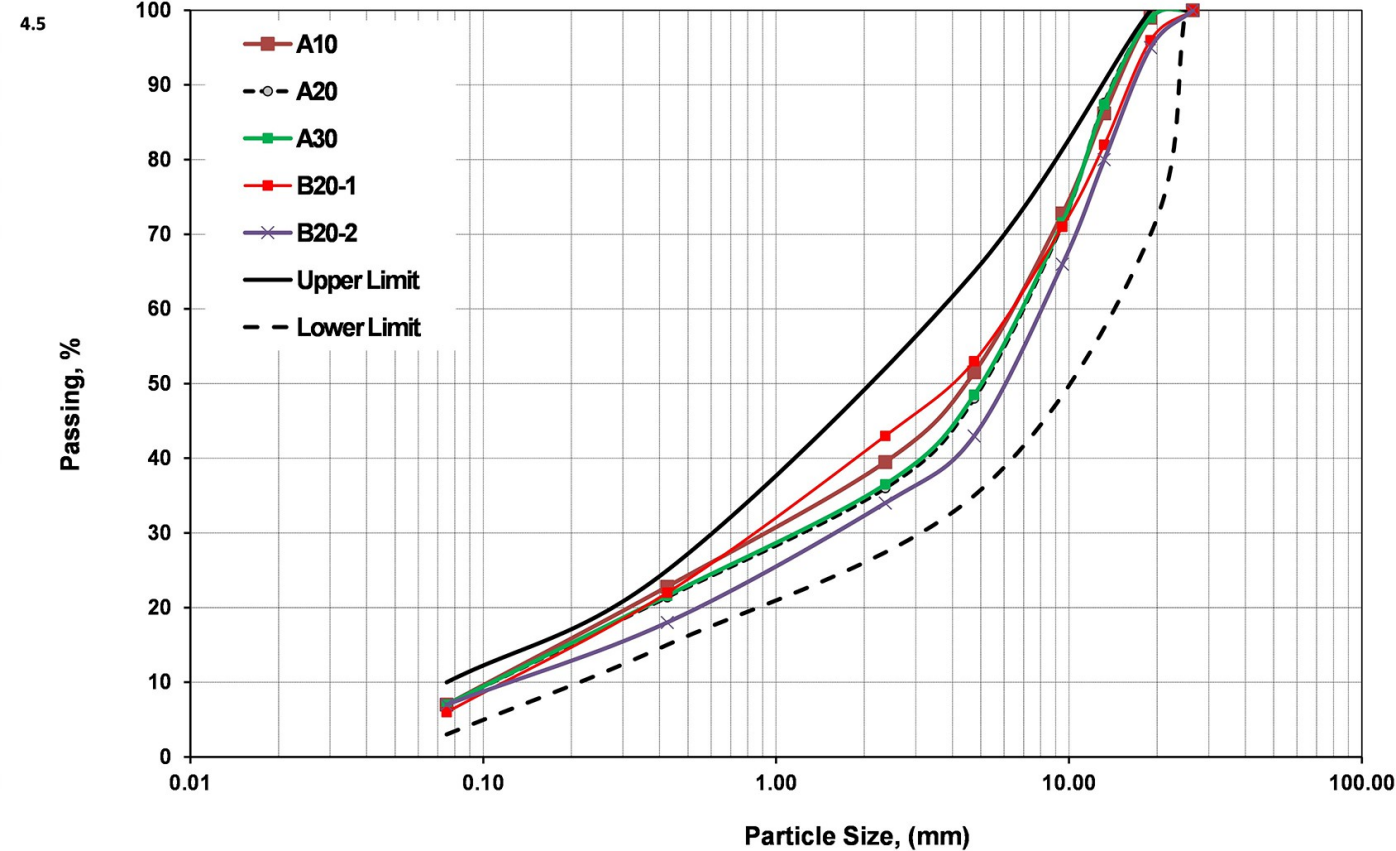
Grading distribution for blends (allowable limits for base-Class 3 are displayed).

The basic engineering characteristics for the different investigated blends compared with the Saudi specifications for base materials are displayed in Table 1. The grading curves for the different blends before compaction are presented in Fig 1 and compared with the limits of the lower and upper specifications for base material (Class 3) as recommended by the Ministry of Transport, Kingdom of Saudi Arabia. It is observed that the grading distribution is within the allowable limits for all blends. It is also observed that the percentage of fines (P#200) is 7% for all blends except B20-1 blend (refer to Table 1). It can be noted from Table 1 that the values of specific gravity in a compliance with the trend of MDD.

Generally, the plasticity of fines, as defined by the plasticity index and liquid limit met Saudi specifications for base materials except for supplier B blends that have liquid limit values of 2% and 1% above the allowable limit for B20-1 and B20-2, respectively. It is obvious from Table 1 that the percentage of brick does not have a significant influence on the liquid limit of fines for the supplier B blends. The LAA values of all blends were within the allowable limit of Saudi specifications for base material which is 45%. It can be noted that the water absorption of the blend increases with the addition of more brick content in the blend.

**Fig 2:**
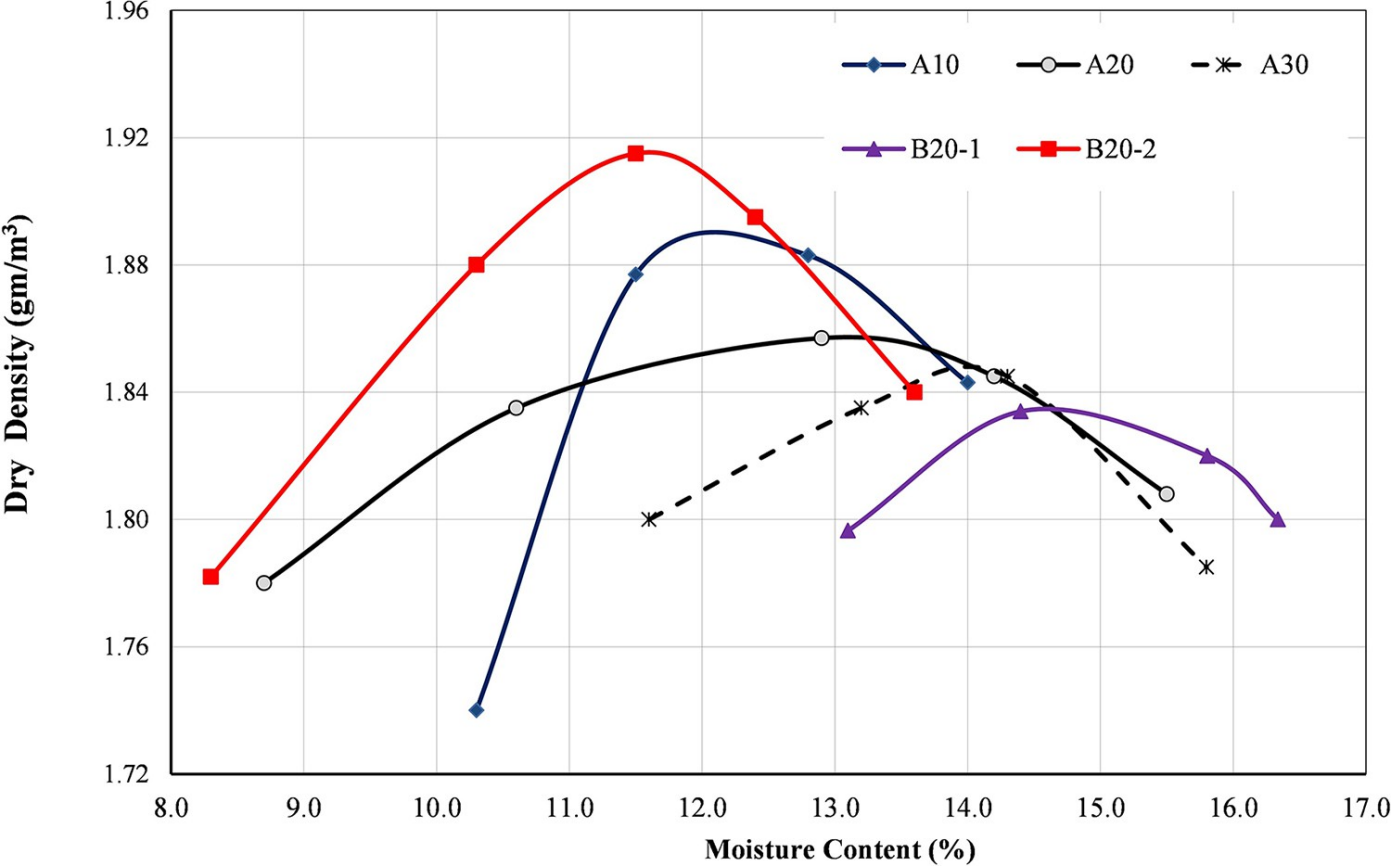
Compaction curves for different materials.

Fig 2 presents the compaction curves for different blends. The values of MDD tend to increase as the replacement level of brick decreases. While the values of OMC in general increase with the increase in the brick content as provided in Fig 2. By comparing the compaction results for B20-1 and B20-2 although they have the same source, B20-2 has high value of MDD and low OMC. This is because materials with a high PI tend to have a lower maximum dry density (MDD) and a higher optimum moisture content (OMC) than soils with a low PI due to the clay particles in plastic soils are more hydrophilic, or water-loving, than the sand particles in non-plastic soils [42]. This observation complies with the results of Poon and Chan [21]. Furthermore, the values of OMC for the different blends in the current study are close to the findings of Arulrajah et al. [1], however, the values of MDD are lower.

**Fig 3:**
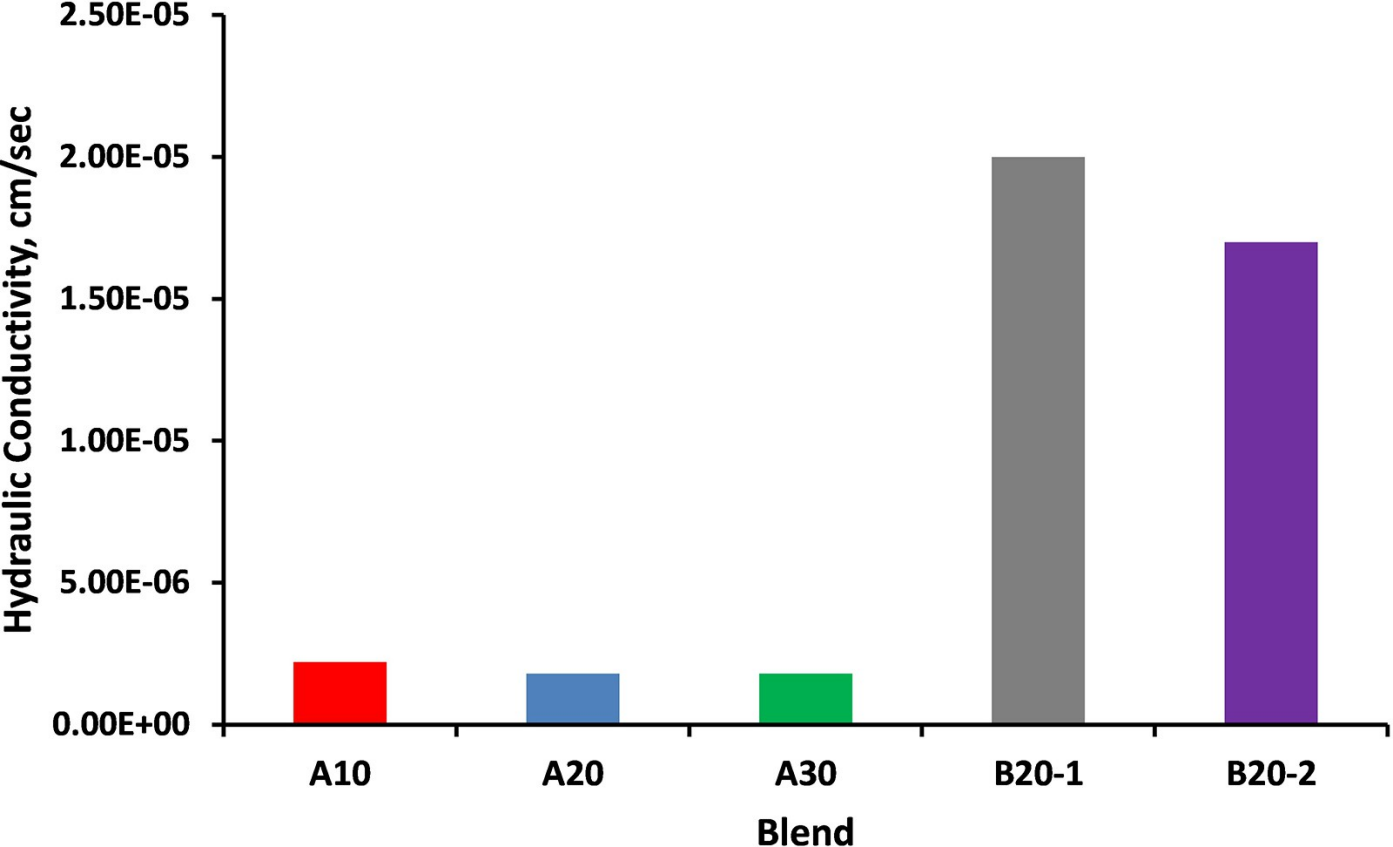
Hydraulic conductivity for different blends.

The hydraulic conductivity for the different materials is illustrated in Fig 3. The findings presented in Fig 3 showed significant differences in hydraulic conductivity between blends from two different suppliers, A and B. The hydraulic conductivity for blends from supplier A is approximately 2x10^-6^ cm/s which indicates a relatively low hydraulic conductivity, meaning that water transmission through these blends is relatively slow. In contrast, blends from supplier B have a hydraulic conductivity of approximately 2x10^-5^ cm/s.

This value is approximately 10 times higher than that of supplier A blends, indicating a significantly higher water transmission rate and it may be due to the low value of PI. Based on the results of hydraulic conductivity, it is concluded that both supplier A and supplier B blends are effective drainage media. This means they have the capacity to facilitate the movement of water, which is a valuable property in many engineering applications, particularly in drainage and geotechnical engineering. The existence of a significant disparity in hydraulic conductivities for materials collected from different stockpiles, even when the materials are of the same type, can be attributed to several factors such as material variability (particle size distribution, mineralogical composition), gradation and particle packing (particle size distribution and packing arrangement of materials) and contaminants and impurities [43–45].

The values of hydraulic conductivity were in close agreement with the findings of Arulrajah et al. and Kruse et al. [1, 45], they found the hydraulic conductivity of recycled brick aggregates ranged from 1x10^-6^ cm/s to 2x10^-5^ cm/s. In general, the findings of the current study are consistent with previous studies, which have shown that the hydraulic conductivity of recycled construction and demolition waste materials can vary depending on the material properties and source of the materials.

The current study investigated the impact of brick replacement level on the CBR values of recycled construction and demolition waste materials (crushed concrete and brick) from two different suppliers (A and B). The findings showed that the CBR values decreased with increasing brick replacement levels which is likely due to the lower strength and durability of brick compared to crushed concrete. The unsoaked CBR values ranged from 125% to 83%, while the soaked CBR values ranged from 112% to 74% for the blends from supplier A. For blends from supplier B, the unsoaked CBR values ranged from 141% to 126%, and the soaked CBR values ranged from 130% to 115%. All the blends achieved the minimum CBR values as required by the Saudi specifications for the construction of the base layer in the local roads. Several studies have investigated the effect of brick replacement level on the CBR values of recycled construction and demolition waste materials. Ahmed et al. [46] found that the CBR values of recycled concrete aggregates decreased with increasing brick replacement levels. Similar findings were reported by Demir et al. [47] and Al-Taie et al. [48].

### 3.2. Static triaxial

#### 3.2.1. Significance of compaction level

**Fig 4:**
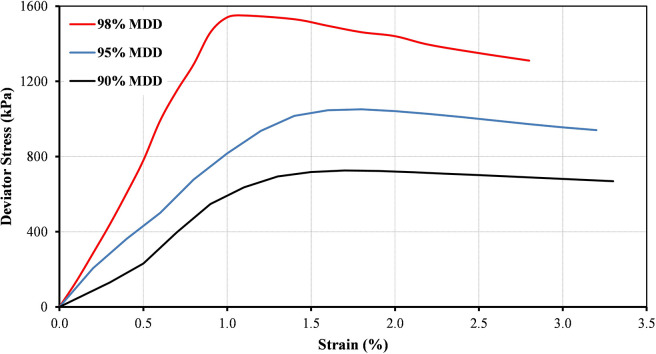
Variations of deviator stress for B20-1 at different compaction levels and at confining pressure of 75 kPa.

**Fig 5:**
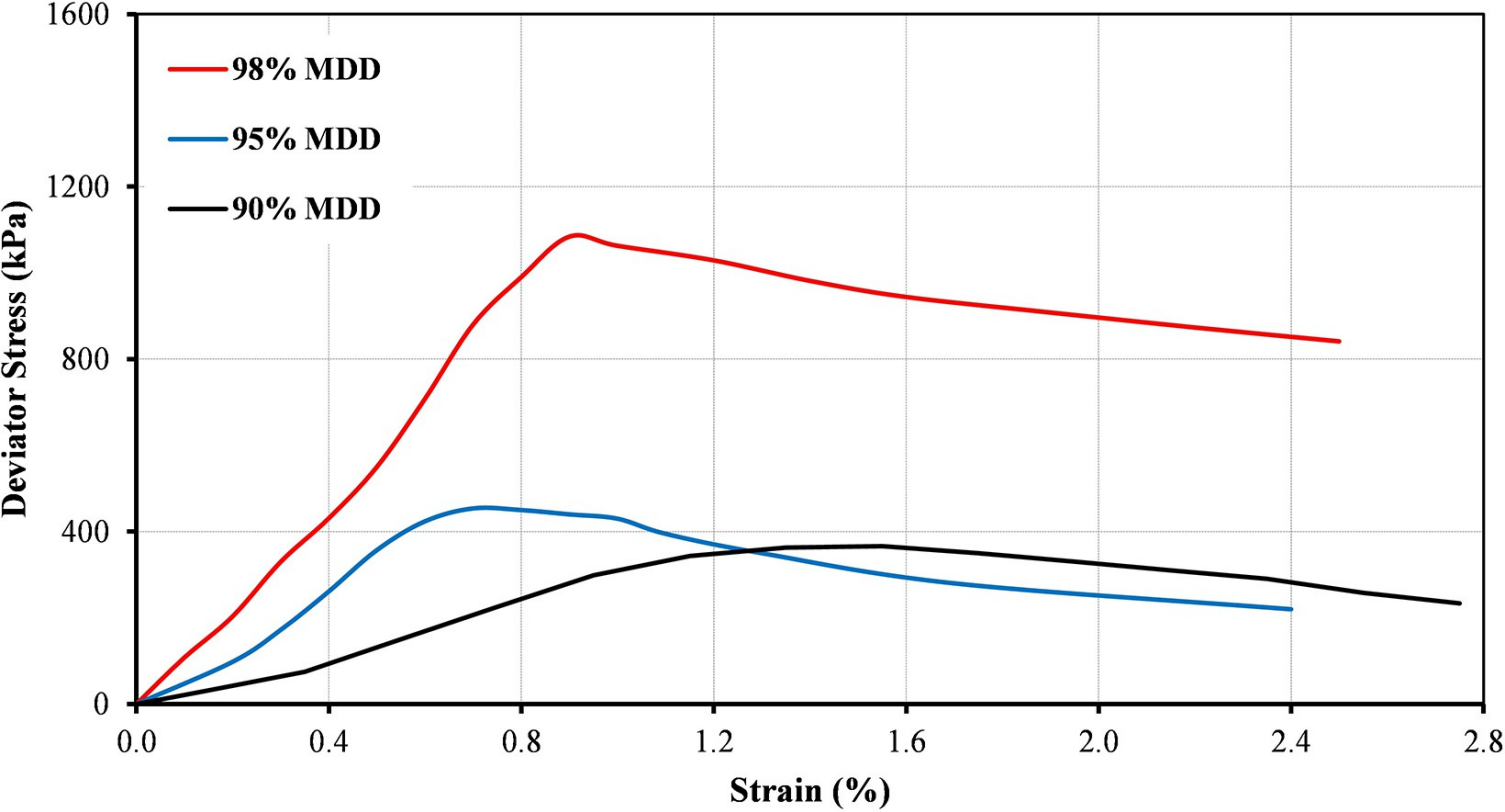
Variations of deviator stress for B20-1 at different compaction levels and at confining pressure of 25 kPa.

The stress-strain curves for the B20-1 blend prepared at different levels of compaction at confining pressures of 75 kPa and 25 kPa as an example are illustrated in Figs 4 and 5, respectively. It can be noted that the peak deviator stress was dependent on the applied confining pressures. It was found that the peak deviator stress increases as the compaction levels increase. The results show some linearity in the relationship at an early stage of low strain.

The shear strength parameters of B20-1 blend at different levels of compaction are provided in Table 2. The data shows that as the compaction level increases, both cohesion and friction angle tend to increase. This is a common trend in granular materials, where higher compaction leads to results in stronger antiparticle bonds and increased material cohesion. The substantial increase in cohesion at the highest compaction level (98%) indicates that the material becomes significantly more resistant to shear forces. The friction angle, on the other hand, responds differently to compaction. While there is a notable increase from 54° to 59° as compaction rises from 90% to 95%, the highest compaction level of 98% returns a friction angle of 55°. This non-monotonic trend could be attributed to various factors, such as particle alignment, dilatancy, and compaction-induced changes in particle interactions (ASTM D4253-16) [49]. It can be concluded that the significance of compaction level on the values of apparent cohesion is clear especially when the value of MDD is higher than 95%. This finding complies with the results of Molenaar and van Niekerk [50]. The values of cohesion and friction angle obtained in the current study at compaction level of 95% within the range of values reported by Al-Taie et al., [48].

**Table 2:** Shear strength parameters for B20-1 blend at different compaction levels and 80% OMC.

*Compaction Level*, *%*	*Cohesion*, *c (kPa)*	*Friction angle*, *ϕ (°)*
90	15	54
95	25	59
98	130	55

**Fig 6:**
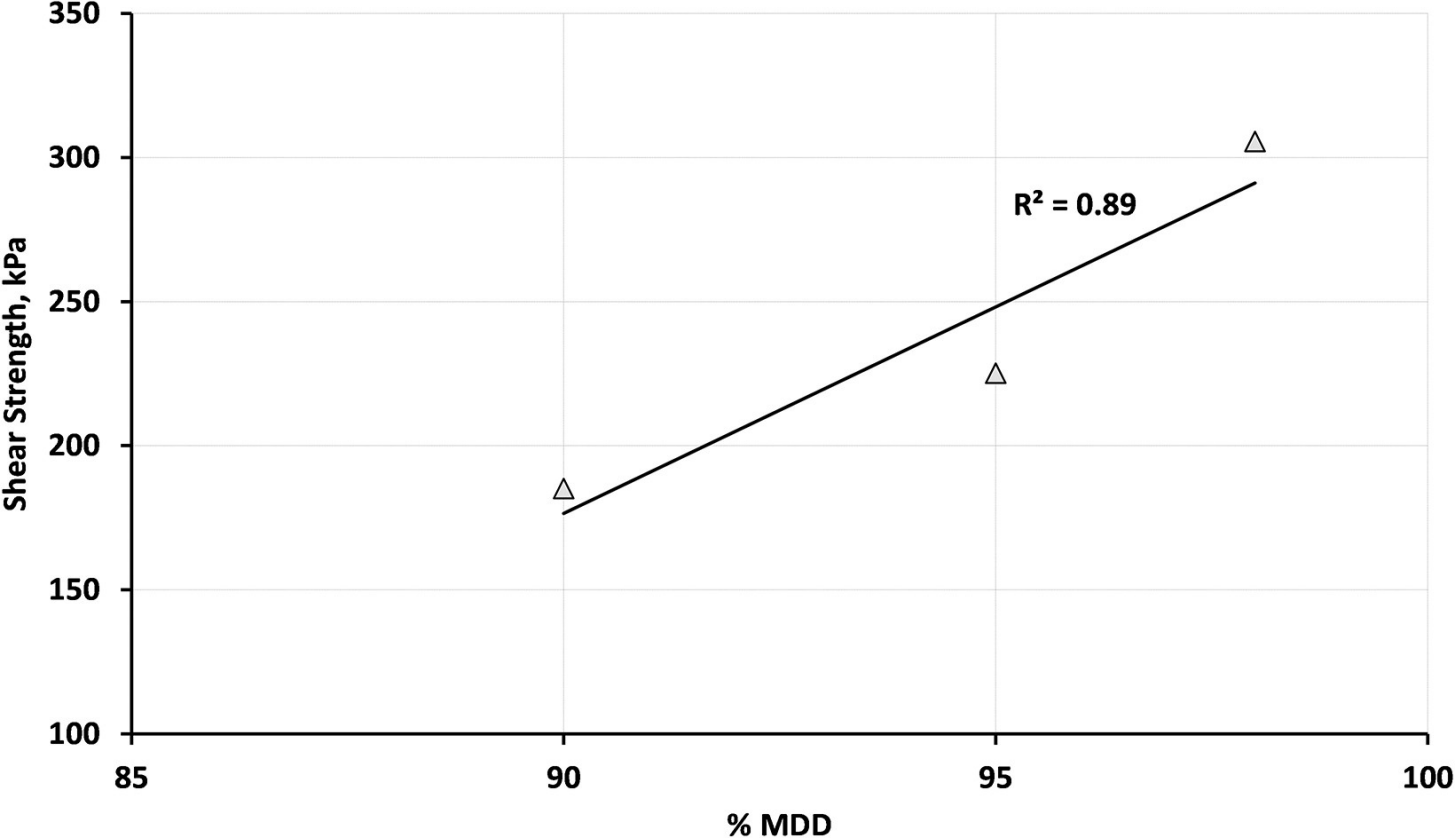
Relationship between shear strength and compaction levels for B20-1 at 80% OMC.

The change of shear strength with the different compaction levels for B20-1 blend is illustrated in Fig 6. The shear strength value was calculated by assuming 150 kPa as the value for normal stress. It can be observed that the shear strength increases with increasing the compaction effort. Also, a linear trend was found between shear strength and compaction levels.

#### 3.2.2. Significance of moisture change

**Fig 7:**
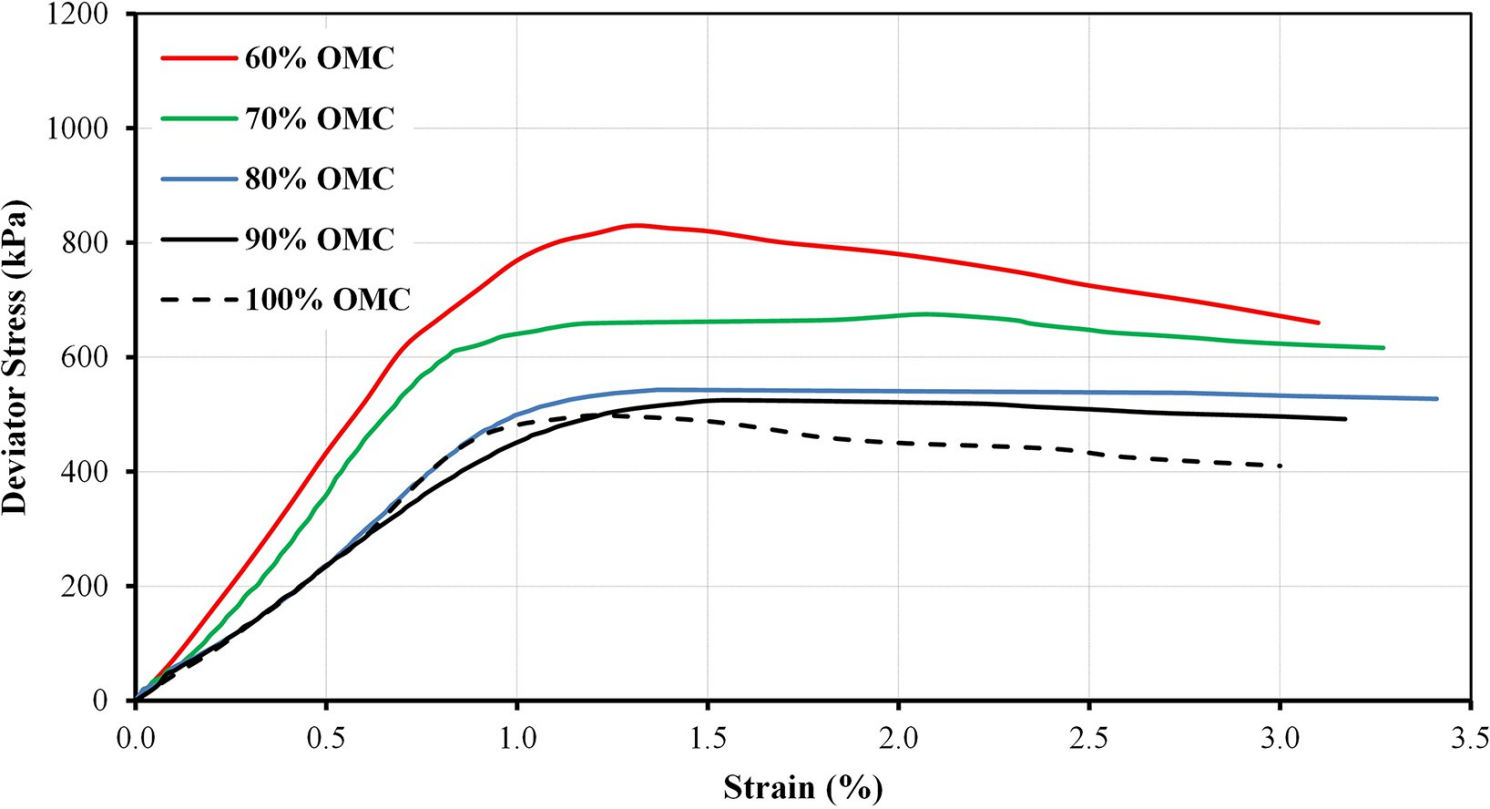
Variations of deviator stress for A20 at different moisture states and at confining pressure of 75 kPa.

**Fig 8:**
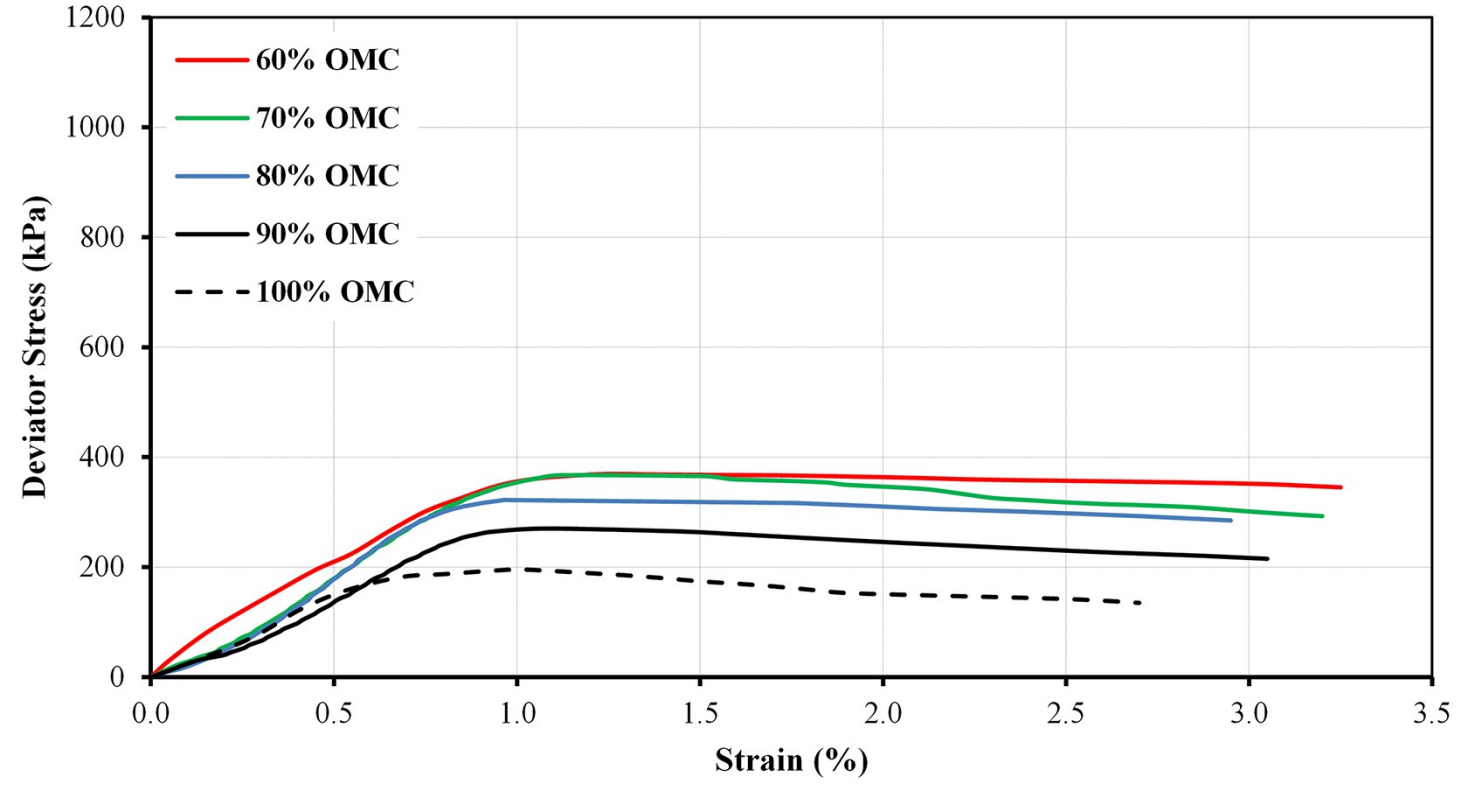
Variations of deviator stress for A20 at different moisture states and at confining pressure of 25 kPa.

The stress-strain curves for the A20 blend prepared at different moisture states and at confining pressures of 75 kPa and 25 kPa as an example are illustrated in Figs 7 and 8, respectively. The stress-strain curves initially exhibit a linear relationship at the early stages of low strain. This linearity indicates that the materials behave in an elastic manner, deforming proportionally to the applied stress. It is noteworthy that the peak deviator stress is dependent on the moisture state. As moisture content decreases, the peak deviator stress increases. This observation shows that drier specimens have a higher shear strength, which is a common behavior for many granular materials. The results clearly indicate that moisture content has a significant influence on the peak deviator stress. This influence may be attributed to the role of water in altering the antiparticle friction and particle-particle interactions within the material. Lower moisture content typically results in a more stable and cohesive material, leading to higher shear strength. It can be seen from Figures that the peak deviator stress values increase as the applied confining pressure rises. This behavior is consistent with the principles of effective stress, which states that as confining pressure increases, the effective stress also increases. As a result, the material exhibits higher peak shear strength under greater confining pressures.

**Fig 9:**
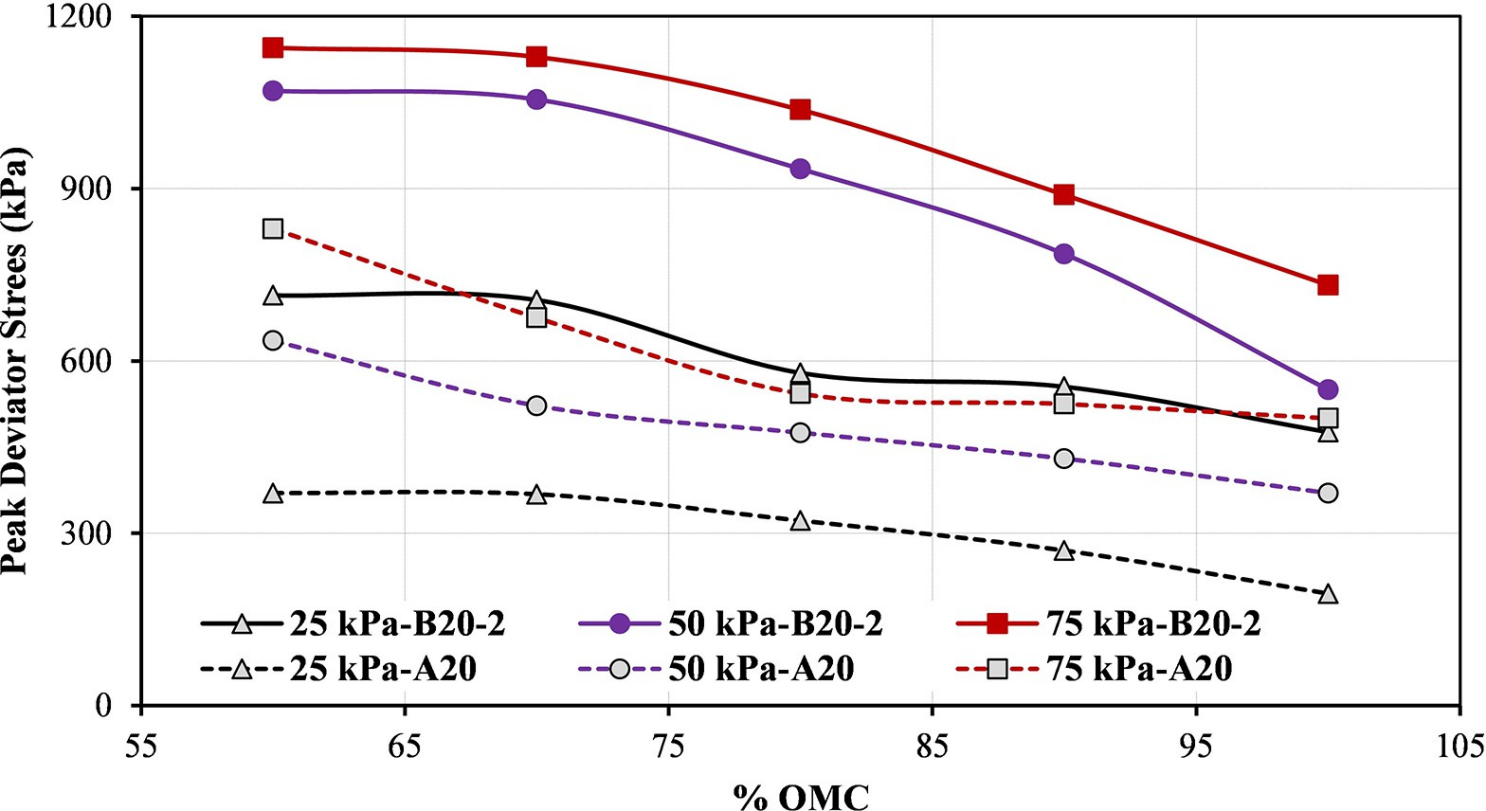
Variations of peak deviator stress for A20 and B20-2 blends at different moisture states and different confining pressure.

The comparison between the two blends, A20 and B20-2 is presented in Fig 9. It indicates that the B20-2 blend consistently exhibits higher peak strength values than A20 blend at different levels of moisture content and confining pressures. This difference may be due to variations in particle size distribution, mineralogy, or compaction characteristics between the two blends. It is essential to consider the specific properties of each blend when assessing their performance.

Table 3 provides the Mohr-Coulomb failure parameters (c and ϕ) for the different blends. It can be noted that there is no consistent trend for the variations of apparent cohesion and friction angle with the change of moisture state. It may be due to the steep slope of the failure envelope for these blends with the small values of the applied confining pressures. The steep slope of the failure envelope means that the shear stress increases rapidly with increasing normal stress which makes the material more likely to fail, especially under high stress conditions. Although of this, there is a clear trend between the shear strength and the variations of moisture state as will be discussed later in this study.

**Table 3:** Shear strength parameters for different blends at 98% MDD.

*Material*	*A10*			*A20*					*A30*			*B20-1*					*B20-2*				
Moisture state, % OMC	70	80	90	60	70	80	90	100	70	80	90	60	70	80	90	100	60	70	80	90	100
Cohesion, c (kPa)	81	9	25	23	40	46	30	9	72	0	32	139	187	134	98	108	89	76	54	70	63
Friction angle, ϕ (°)	41	53	51	55	49	44	46	49	37	53	45	60	51	55	61	60	53	56	56	51	47

**Fig 10:**
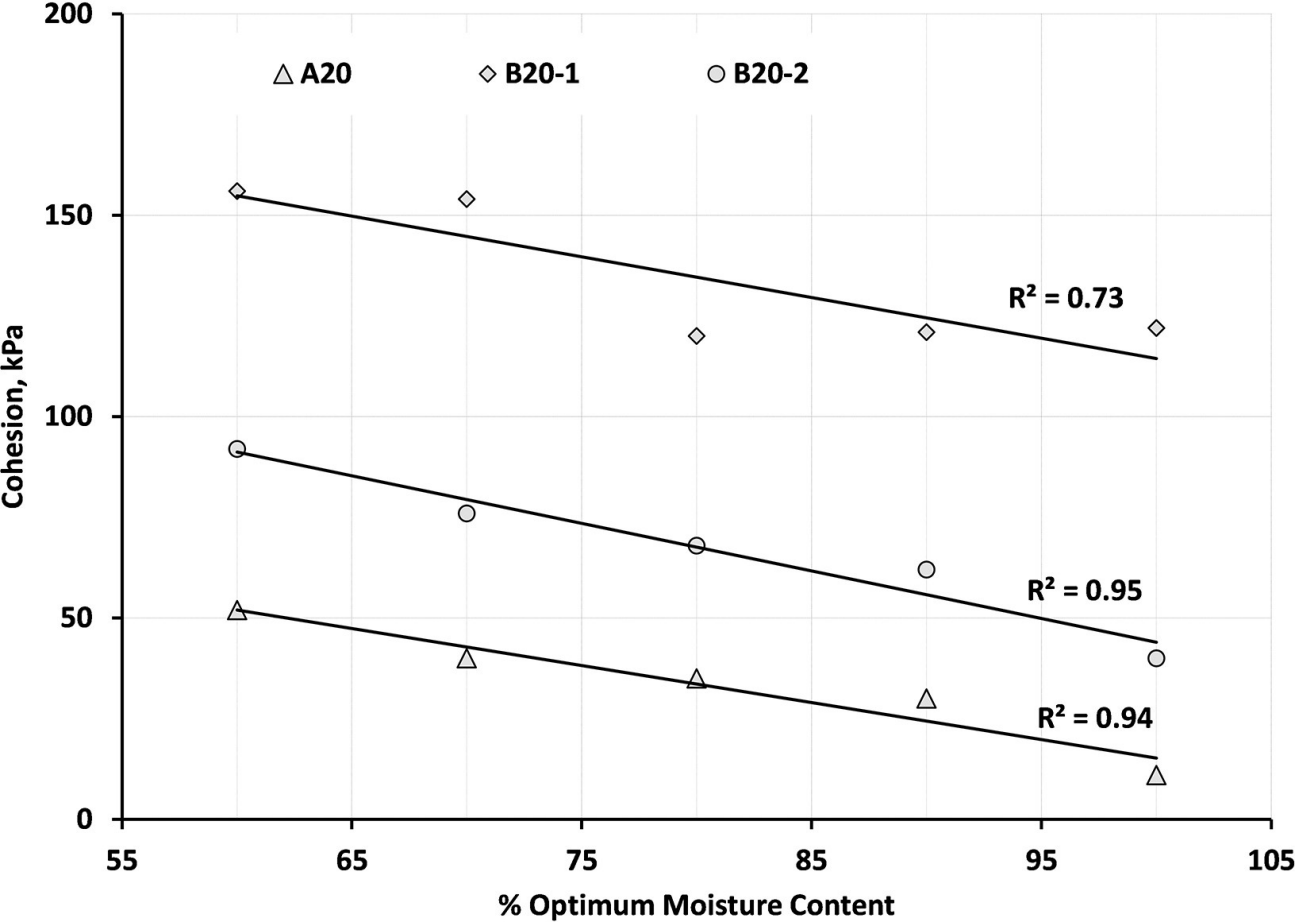
Relationship between apparent cohesion and variations of moisture state for A20, B20-1, and B20-2 blends.

The effect of suction on cohesion is particularly pronounced at intermediate moisture contents. At these moisture levels, the capillary forces created by suction can effectively bridge between soil particles, creating attractive forces that further enhance the soil’s resistance to shearing. This is why unsaturated soils often exhibit a peak shear strength at intermediate moisture contents [42, 51]. At very low moisture contents, the shear strength of the soil is primarily determined by its internal friction, which is a resistance to shearing due to the interlocking of soil particles. As moisture content increases, soil suction begins to play a more significant role in resisting shearing. This is because suction contributes to the cohesion of the soil, which is an additional resistance to shearing independent of the normal stress. It is also reported for the compacted material at different levels of moisture state but at the same compaction level, the strength will change because of the difference in soil suction differences, [52, 53], the friction angle was assumed to be the same for each material. Consequently, the mean angle friction for each material was determined, and accordingly, the apparent cohesion intercept was corrected. The adjusted cohesion values against the variations of moisture state for A20, B20-1, and B20-2 blends have been plotted in Fig 10. In general, as the moisture state of the sample increases the apparent cohesion is observed to decrease.

**Fig 11:**
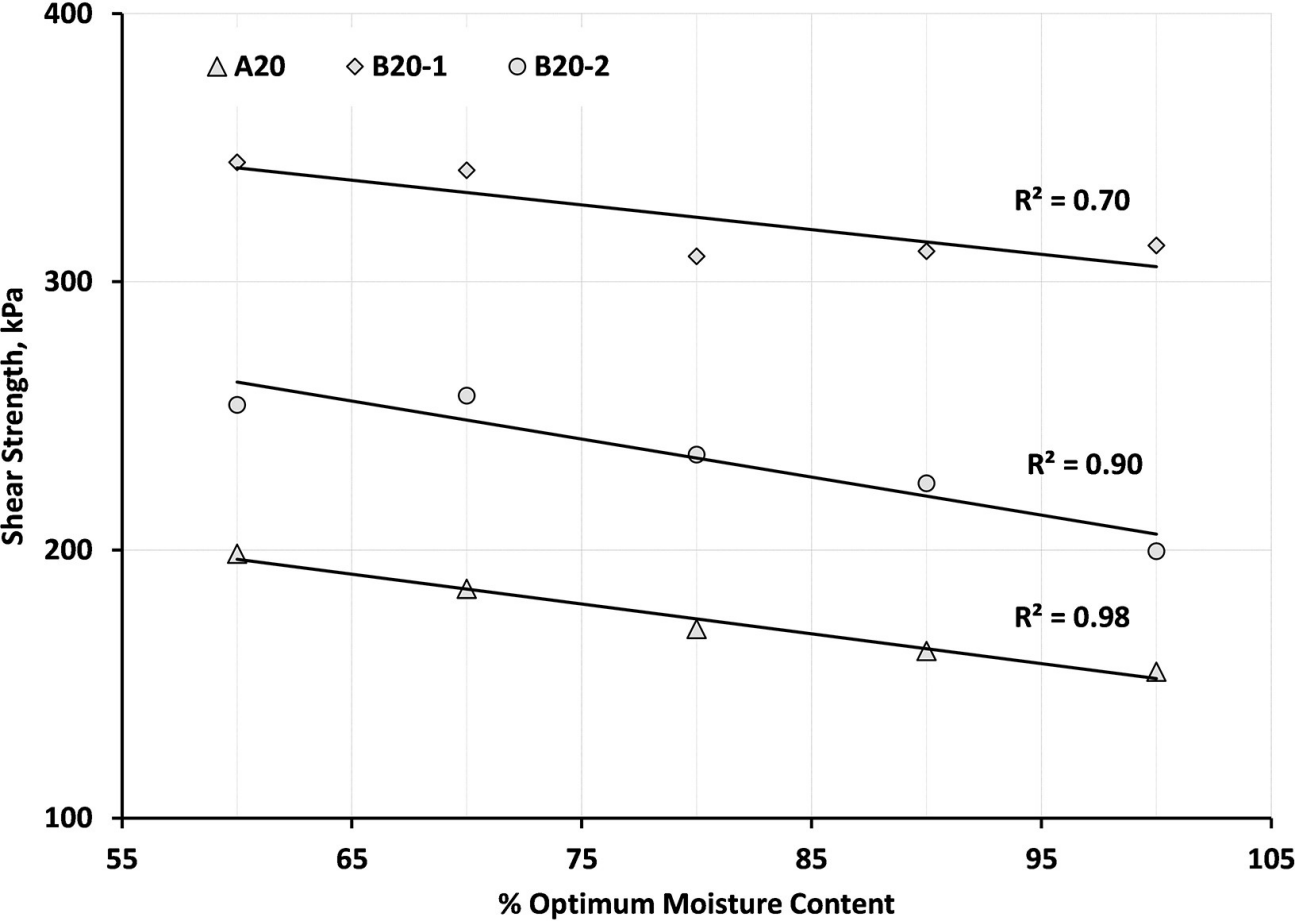
Relationship between shear strength and moisture state for A20, B20-1, and B20-2 blends.

The variations of shear strength with changing the moisture state for A20, B20-1, and B20-2 blends are illustrated in Fig 11. The shear strength value was calculated by assuming 150 kPa as the value for normal stress. As expected, a linear relationship was found between shear strength and moisture state.

### 3.3. Particle breakage

The influence of particle breakage on the properties of granular materials can vary depending on the specific application. It is important to recognize that particle breakage can have both positive and negative effects. Particle breakage can lead to a redistribution of particles within a material, creating a denser and better-compacted structure [43, 54]. Smaller, rounded particles resulting from breakage may improve the packing of the material, reducing void spaces and increasing the material’s resistance to shear deformation (ASTM D6938) [55]. On the other hand, excessive particle breakage can lead to changes in the material’s particle size distribution and reducing particle-to-particle contact and interlocking (ASTM D4253) [49]. Furthermore, the particle breakage can changes in particle shape and can affect dilatancy behavior [43].

#### 3.3.1. Significance of compaction level

**Fig 12:**
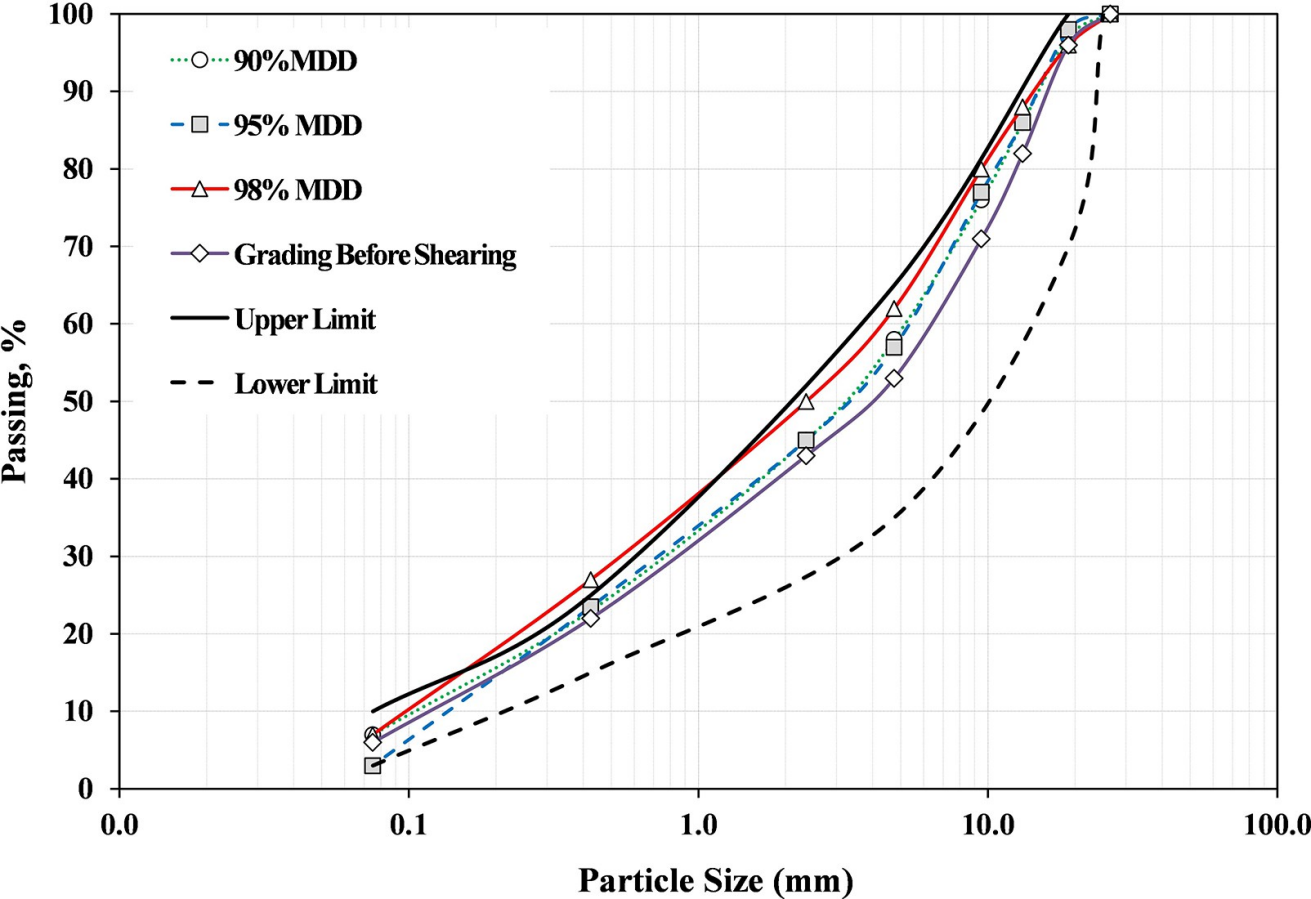
Grading distribution for B20-1 blend prepared at 80% OMC and different compaction levels (after compaction).

The difference between the grading before compaction and after compaction and shearing is defined as particle breakage. Fig 12 shows the grading distribution for B20-1 blend prepared at 80% OMC and different compaction levels. It can be observed from Fig 12 that the grading was significantly changed after compaction, and shearing, and moved generally around the upper grading limit. This observation is in agreement with the findings of Gabr [56]. Also can be noted that grading at 98% MDD becomes finer through all particle sizes compared to other compaction levels. It is due to the application of high compressive forces during the compaction process of specimens and shearing that resulted in particle breakdown. When comparing the grading curves after compaction and shearing with the Saudi specifications, it can be seen that the grading of all blends is still within the limits except for the percentage of passing through sieve #40 (refer to Fig 12).

#### 3.3.2. Significance of moisture change

**Fig 13:**
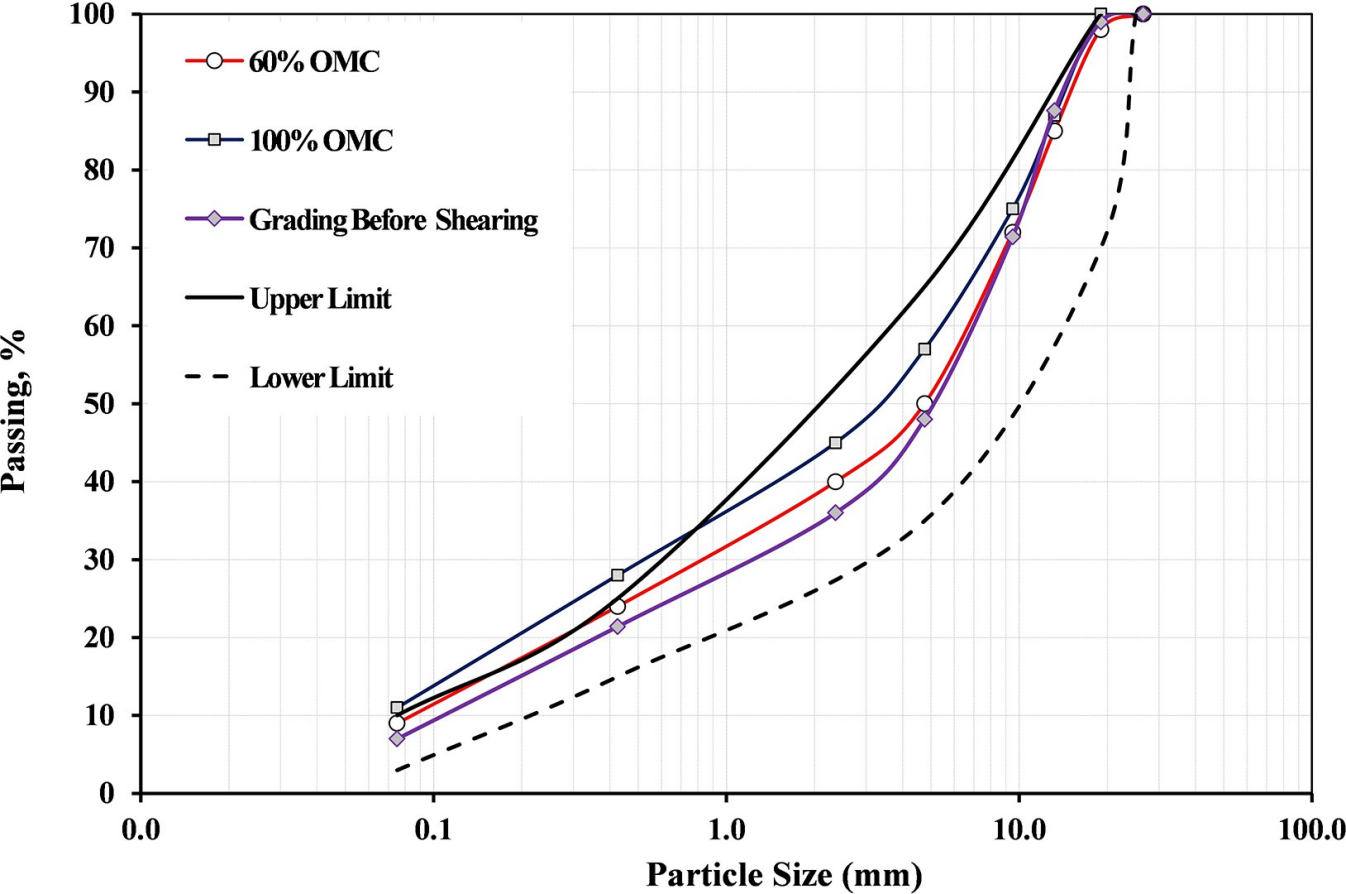
Grading distribution for A20 blend prepared at different moisture content and 98% MDD (after compaction).

Fig 13 provides an example of the grading curves for the material A20 after shearing at the moisture content of 60% and 100% of OMC and 98% MDD compared with grading before shearing. It can be seen from the figure the blend becomes more fine compared with the original grading (before compaction), especially for the particle size ≤ 4.75 mm. It is also noted that the percentage of fines (passing #200) increased from 7% to 11%. It is worth noting that as the sample moisture content increases, the grading becomes finer as the moisture made the particles of the blend easy to crush under the application of loading. The grading curves after shearing are still within the limits except for the sample prepared at 90% OMC which has the passing from sieve sizes 0.075 mm and 0.425 mm out of specifications (refer to Fig 13).

**Fig 14:**
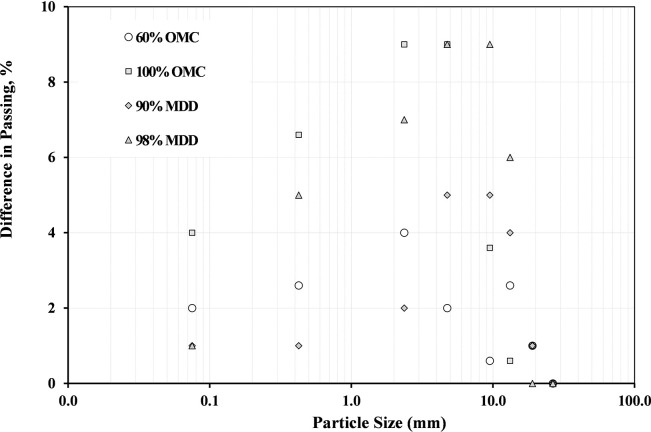
Difference in grading distribution for A20 blend prepared at different moisture content and MDD (after compaction).

Fig 14. shows that the difference in grading distribution for A20 blend prepared at different moisture content and MDD (after compaction). It can observed from the figure that difference in passing increases with increasing particle size and decreasing OMC and MDD. The difference in passing is greatest at the smallest particle size (0.01 mm) and at the lowest OMC/MDD (60%). This is because the smallest particles are most likely to be retained on the sieve and soils with lower OMC/MDD are more likely to have a higher void ratio. It is also noted that the difference in passing decreases with increasing OMC/MDD due to the soil particles are more tightly packed together at higher OMC/MDD, leaving less space for larger particles to fit through the sieve. The figure also shows that the difference in passing is greatest for the 98% MDD curve. This is because the 98% MDD curve represents the densest possible state of the material. In this state, the soil particles are tightly packed together, leaving very little space for larger particles to fit through the sieve. Based on the results of this figure, it is important to consider the particle size distribution and OMC/MDD of the soil when selecting a sieve size for gradation testing.

## 4. Conclusions

The basic engineering characteristics of two different products were assessed in this study. Materials were evaluated according to their simple and complex engineering characteristics. An evaluation of the sensitivity of shear strength and particle breakage to moisture state and compaction level was conducted. Using the results of this study, the basic engineering characteristics of the different blends met the Saudi specifications for road construction as a base material. With increasing replacement levels of brick in the blend, CBR values decreased. It was found that he unsoaked CBR values ranged from 125% to 83%, while the soaked CBR values ranged from 112% to 74% for the blends from supplier A. The hydraulic conductivity for blends from supplier A is approximately 2x10^-6^ cm/s, which indicates a relatively low hydraulic conductivity, meaning that water transmission through these blends is relatively slow. It is obvious that the A supplier blend has almost the same hydraulic conductivity (2x10^-6^ cm/s). In contrast, blends from supplier B have a hydraulic conductivity of approximately 2x10^-5^ cm/s. Furthermore, a reverse linear relationship was found between the change in moisture state and apparent cohesion and shear strength. Shear strength and compaction levels, however, showed a proportional trend. It is worth noting that the shear strength of blends will increase with increased compaction effort. Additionally, as the sample moisture content and compaction level increase, particle breakage increases, and the grade becomes finer as the moisture made the particles of the blend easier to crush. Therefore, the significance of compaction effort is greater than the change in moisture state as the compacted soil is more stable and can support more weight without collapsing [36]. Moisture change, on the other hand, can have both positive and negative effects on soil. On the positive side, moisture can help to bind soil particles together, making the soil more stable. Additionally, moisture can help to lubricate soil particles, making it easier for them to slide past each other. However, too much moisture can also have negative effects on soil. Excess moisture can make the soil more susceptible to erosion and landslides. The compaction has a more permanent effect on the soil. Once soil is compacted, it will remain compacted until some external force disturbs it. Moisture change, on the other hand, is a temporary condition that can be reversed by changes in the weather.

If construction and demolition waste is employed as a base layer, strict control should be exercised over the compaction process. During construction, compaction is relatively easy to achieve and is relatively low cost, resulting in pavement materials being stiffer.

The findings of this study hold significant practical implications for the use of construction and demolition waste in road construction projects. Therefore, the practical applications of this study extend to the engineering and construction industry, where the use of recycled construction and demolition waste can be a sustainable and economically viable choice for road construction projects. By understanding the material-specific behavior and the factors that influence its engineering properties, engineers and construction professionals can make informed decisions that promote the efficient use of recycled materials, reduce waste, and enhance the overall sustainability of road infrastructure development. The current study focused on two specific types of recycled construction and demolition waste materials (crushed concrete and brick). It would be beneficial to expand the study to include a wider range of recycled materials to assess their suitability for road construction applications. It is also primarily investigated the basic engineering characteristics of the recycled materials and their relationship to moisture state and compaction level. Further research is require to evaluate the long-term performance of these materials under various traffic loading and environmental conditions. It is also recommended to conduct economic and environmental life cycle assessments to compare the sustainability of using recycled construction and demolition waste materials in road construction with conventional materials. Finally, tt is recommended to have strict control over the compaction process of construction and demolition waste when employed as a base layer. It is relatively easy to achieve high levels of compaction during the construction process at relatively low costs which resulted in improving the stiffness of pavement materials.
